# Sex differences in the kinematics and kinetics of the foot and plantar aponeurosis during drop-jump

**DOI:** 10.1038/s41598-023-39682-6

**Published:** 2023-08-10

**Authors:** Yuka Matsumoto, Naomichi Ogihara, Sachiko Kosuge, Hiroki Hanawa, Takanori Kokubun, Naohiko Kanemura

**Affiliations:** 1 Department of Biological Sciences, The University of Tokyo, Tokyo, Japan; 2 Graduate Course of Health and Social Services, Graduate School of Saitama Prefectural University, Saitama, Japan; 3 Maeda Seikeigeka, Saitama, Japan; 4 Department of Health Science, University of Human Arts and Sciences, Saitama, Japan; 5 Department of Health and Social Services, Saitama Prefectural University, 820 Sannomiya, Koshigaya, Saitama 343-8540 Japan

**Keywords:** Anatomy, Engineering

## Abstract

Plantar fasciitis is one of the most common musculoskeletal injuries in runners and jumpers, with a higher incidence in females. However, mechanisms underlying sex-associated differences in its incidence remain unclear. This study investigated the possible differences in landing and jumping kinematics and kinetics of the foot between sexes during drop-jump activities. Twenty-six participants, including 13 males and 13 females, performed drop-jumps from a platform onto force plates. Nineteen trials including ten males and nine females were selected for inverse dynamics analysis. The patterns of stretch and tensile force generated by the plantar aponeurosis (PA) were estimated using a multi-segment foot model incorporating the PA. Our results demonstrated that dorsiflexion, angular velocity, and normalized plantarflexion moment of the midtarsal joint right after the heel landed on the floor were significantly larger in females than in males. Consequently, the PA strain rate and tensile stress tended to be larger in females than in males. Such differences in the kinematics and kinetics of the foot and the PA between sexes could potentially lead to a higher prevalence of foot injuries such as plantar fasciitis in females.

## Introduction

Plantar fasciitis, a degenerative inflammation of the plantar aponeurosis (PA) caused by excessive and repetitive mechanical stress^1,2^, is one of the most common musculoskeletal injuries in runners and jumpers, along with medial tibial stress syndrome, Achilles tendon injury, and patellofemoral pain syndrome^3,4^. Plantar fasciitis reportedly accounts for approximately 5–10% of all running-related injuries^5–11^. Repeatedly jumping and landing on the feet is also known to be a high-risk activity of plantar fasciitis^12–16^. Approximately 10% of the population is expected to experience heel pain due to plantar fasciitis in their lifetime^17^, causing difficulties in running and other sports and recreational activities.

There is a widespread perception that females are more commonly affected by plantar fasciitis than males^13,18–29^. It was reported that 59.8% of approximately 800,000 patients diagnosed with plantar fasciitis in the United States were female^23^. It was also reported that the incidence rate of plantar fasciitis was approximately two times larger in females than in males among the members of the United States military^20^. A study based on a health survey of 75,000 participants showed that females were 2.5 times more likely to be diagnosed with plantar fasciitis than males in the adult United States population^29^. However, the mechanism underlying this observed sex-related difference is not well documented in the current literature.

It has recently been suggested that midfoot motions during dynamic movements such as walking, running, and jumping are significantly larger in females than in males^30–34^, possibly due to sex differences in foot bone morphology^35–38^ and joint laxity of the foot^34,39^. If this is the case, the patterns of stretch and force generation of the PA during dynamic movements, and hence those of PA strain rate and tensile stress might differ between sexes, and this could be a possible reason why the prevalence of plantar fasciitis is higher in females than in males. However, direct in vivo measurements of the strain and tensile stress generated by the PA during movements are technically impossible without using invasive techniques. Hence it remains unclear why the prevalence of plantar fasciitis is higher in females than in males.

In this study, we investigated the possible differences in kinematics and kinetics of the foot, as well as the pattern of stretch and force generation of the PA between sexes during drop-jump using a multi-segment foot model incorporating the PA. Specifically, we tested the hypothesis that the strain rate and tensile stress of the PA were larger in females than in males during drop-jump. A drop-jump is a vertical jump wherein participants drop down from a platform and then jump upward as high as possible with rapid ground contact time. We investigated drop-jump because sex-associated differences in the kinematics and kinetics of the foot and PA might be more clearly observed in drop-jump than in other dynamic movements, such as walking and running because of larger ground reaction force applied to the body^40,41^.

## Methods

### Participants

Thirteen male and thirteen female university students or instructors aged 20 to 40 years who performed no regular training or sports were recruited to participate in the present study since we hypothesized that the prevalence of plantar fasciitis was higher in females than in males because of the differences in kinematics and kinetics of the foot and PA between sexes before the onset of plantar fasciitis. Participants who had any history of foot or lower limb orthopedic, neurological, or musculoskeletal disorders likely to affect the performance of drop-jumps were excluded from the experiment. Participants who had an obvious deformity of the foot such as *pes cavus* and *pes planus* were also excluded. The number of participants was determined by referring to previous studies^31,32^. In accordance with the Declaration of Helsinki, all participants provided written informed consent following a detailed explanation of the study’s purpose and risks. The experimental procedure for this study was approved by the Ethics Committee on Human Experimentation at Saitama Prefectural University (No. 29508). All methods were performed in accordance with the relevant guidelines and regulations.

## Experimental procedure

**Figure 1:**
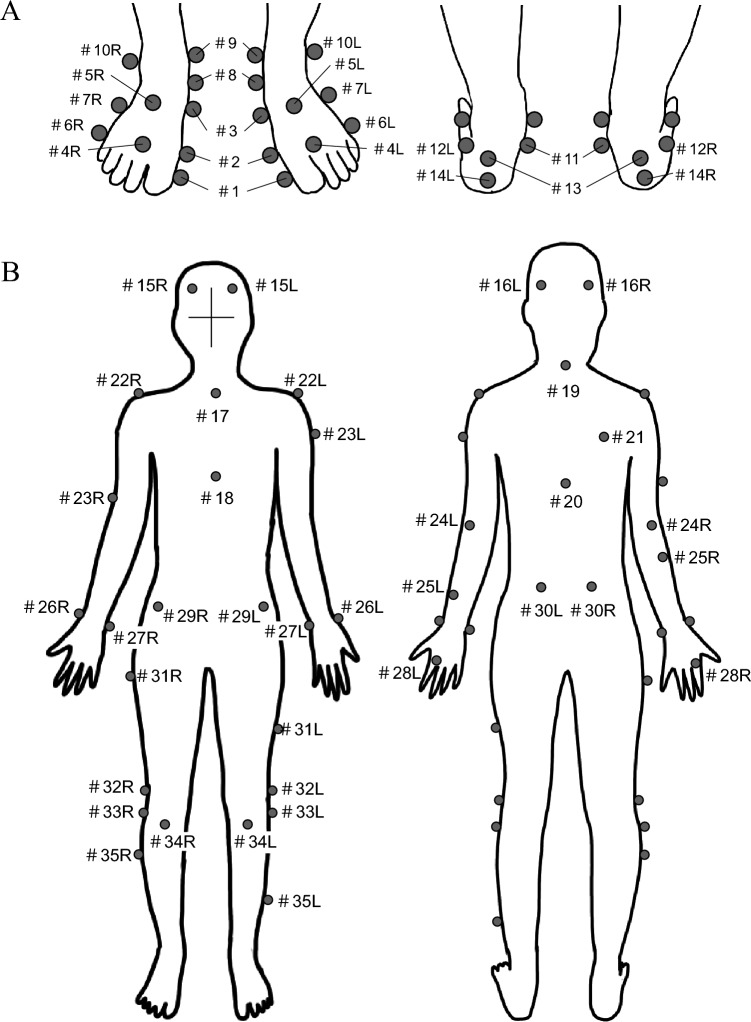
Marker placement of the foot (**A**) and whole body (**B**). R, right; L, left.

In this study, we applied a multi-segment foot model incorporating the PA^42^ for detailed kinematic analysis of the foot during drop-jump. The foot model comprised three segments (phalanx, forefoot, and hindfoot) and five linear springs (PA1–5, from medial to lateral) representing the PA connecting the origins and the insertions via intermediate points defined on the hindfoot, phalanx, and forefoot, respectively, based on the CT data of an adult human foot^42^. For this, 28 infrared-reflective markers (diameter 9.5 mm; 14 per side) were attached to the foot. The foot model was scaled based on the attached markers to create a subject-specific model, which was used to quantify the foot’s intersegmental kinematics and the PA’s elongation pattern during the drop-jump. An additional 37 markers (diameter 14 mm) were placed on the rest of the body surface to capture the whole-body kinematics in accordance with the Plug-in-Gait Full-body AI model^43,44^ (Table 1 and Fig. 1).

**Table 1:** Definition of marker placement.

Number	Description
#1	Dorso-medial aspect of the first proximal phalanx head
#2	Dorso-medial aspect of the first metatarsal head
#3	Dorso-medial aspect of the first metatarsal base
#4	Dorso-medial aspect of the second metatarsal head
#5	Dorso-medial aspect of the second metatarsal base
#6	Dorso-lateral aspect of the fifth metatarsal head
#7	Dorso-medial aspect of the first metatarsal base
#8	Most medial apex of the navicular bone
#9	Distal apex of the medial malleolus
#10	Distal apex of the lateral malleolus
#11	Most medial apex of the sustentaculum tali
#12	Lateral apex of the peroneal tubercle
#13	Apex of the calcaneal tuberosity
#14	Superior apex of the calcaneus
#15	Front of head, above the temple
#16	Back of head, roughly in a horizontal plane of front head (#15) markers
#17	Jugular notch where the clavicles meet the sternum
#18	Xiphoid process of the Sternum
#19	Spinous process of the 7^th^ cervical vertebrae
#20	Spinous process of the 10^th^ thoracic vertebrae
#21	Middle of the right scapula
#22	Acromio-calvicular joint
#23	Lower (right) or upper (left) arm between the shoulder (#22) and elbow (#24) markers
#24	Lateral epicondyle approximating elbow joint
#25	Upper (right) or lower (left) forearm between the elbow (#24) and finger (#28) markers
#26	Radial styloid
#27	Ulnar styloid
#28	Dorsum aspect of the head of the second metacarpal
#29	Anterior superior iliac spine
#30	Posterior superior iliac spine
#31	Lateral upper (right) or lower (left) 1/3 surface of the thigh
#32	Lateral epicondyle of the knee
#33	Head of fibula
#34	Tibial tuberosity
#35	Lateral upper (right) or lower (left) 1/3 surface of the shank

**Figure 2:**
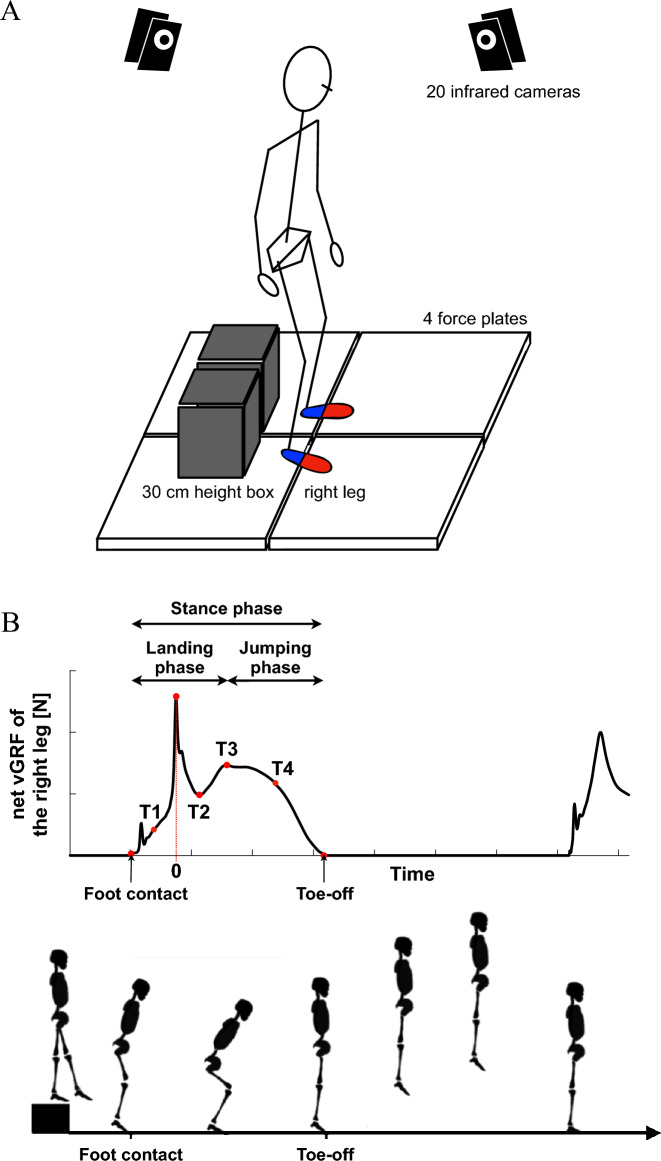
Experimental setup (**A**) and a typical vertical ground reaction force profile of a drop-jump (**B**). Participants were asked to drop down from a platform (30 cm high) and then jump upward as high as possible with rapid ground contact time. The time of impact peak was defined as *t* = 0. T1, T2, T3, and T4 indicate the midpoint between foot contact and the impact peak, the minimum point after the impact peak, the second peak where the vertical position of the COM was minimum, and the midpoint between T3 and toe-off. T1 and T3 are in the landing phase, whereas T4 is in the jumping phase.

Participants performed a drop-jump on four force plates (two per side) adjacent to each other (Fig. 2), and the foot kinematics and ground reaction forces (GRFs) were measured. Participants were instructed to drop down from a platform (30 cm high) and then jump upward as high as possible with rapid ground contact time. To descend from the platform, they were instructed to step off with their right leg while their left leg remained on the platform (step-off technique^45–48^) (Fig. 2B). In addition, they were instructed to land on both anterior and posterior force plates with their fore- and hind-foot, respectively. The movement of the upper limb was not constrained in this study. The distance between the platform and the force plates was adjusted prior to measurement so that the fore- and hind-foot naturally landed on the front and rear force plates, respectively. Each participant performed at least five drop-jump trials at a comfortable interval with sufficient rest after a suitable practice. Marker trajectories were collected using a motion analysis system (Vicon Nexus 2.10.2, Vicon, Oxford, UK) with 20 infrared cameras at 100 Hz. The GRFs were collected using four force plates (9287C, Kistler Instrumente AG, Winterthur, Switzerland) at 1000 Hz.

### Data selection

The drop jump using the step-off technique is essentially asymmetric, with the larger force is known to be applied to the leading leg^48^. This study analyzed the kinematics and kinetics of the right leg. We selected a typical single trial for each participant that satisfied the following criteria for further analysis: (i) the fore- and hind-foot successfully landed on the front and rear force plates, respectively (the navicular and the fifth metatarsal base markers were located within the range of ± 15 mm from the edges of the two force plates at heel-contact), (ii) the heel firmly contacted the ground during landing (as whether the heel contacted the ground or not largely affected the GRF profiles), and (iii) the contact time was < 0.3 s (as we instructed participants to jump as quickly as possible).

#### Definition of phases and time alignment

The stance phase was defined as the period when the net vertical GRF (vGRF) exceeded 2% of each participant’s body weight (BW). The typical vGRF profile exhibits an impact peak soon after the foot contacts the ground. The impact peak basically corresponds to heel-contact. After the impact peak diminishes, the net vGRF profile reaches the second peak when the whole-body center of mass (COM) reaches the minimum vertical position (Fig. 2B). Then the net vGRF decreases as the COM moves upward. Therefore, the early stance phase from the foot contact to the second peak is defined as the landing phase, and the late stance phase from the second peak to the toe-off is defined as the jumping (push-off) phase (Fig. 2B)^48^. For kinematic and kinetic data comparisons, the time is usually normalized by the stance phase duration as in Wilder et al.^48^. However, since the timings of the two peaks were quite variable across the participants, we did not normalize the time, but instead aligned the data with the impact peak (*t* = 0) for comparisons.

Therefore, to compare the differences in the kinematic and kinetics of the foot and PA between sexes, we defined the following four-time points based on the vGRF profile (Fig. 2B): (T1) the midpoint between foot-contact and the impact peak, (T2) the minimum point after the impact peak, (T3) the second peak, and (T4) the midpoint between T3 and toe-off. T1 and T3 were in the landing phase, whereas T4 was in the push-off phase (Fig. 2B).

### Data processing

Marker trajectories were filtered using a fourth-order zero-phase low-pass Butterworth filter with a cutoff frequency of 24 Hz. The GRF profiles were not filtered. The joint angles, moments, and powers of the metatarsophalangeal (MTP), midtarsal, and ankle joints were calculated as described by Matsumoto et al.^42^ using Euler angles and based on inverse dynamics. The angles, moments, and powers of the proximal joints (knee and hip joints) were calculated using Vicon Nexus 2.10.2. (Vicon, Oxford, UK) in the same manner. The joint angles were zero when the joints were in quiet standing. The joints angles were positive for dorsiflexion, flexion, eversion, and abduction. The GRFs were normalized by BW. The joint moments of the foot were normalized by BW and foot length (between the heel and toe markers), and those of the leg were normalized by BW and leg length (between the anterior superior iliac spine and the medial malleolus markers) with reference to Kadaba et al.^49^.

The changes in the PA length, velocity, and force were calculated as described by Matsumoto et al.^42^. Briefly, the subject-specific model was used to calculate the time changes in the positions of the origin, insertion, and intermediate points defining the PA lengths from the motion-captured marker coordinates during drop-jump. The strain of the PA was calculated assuming that the natural length of the PA was approximately 0.98 times the PA length during quiet standing^42^. To quantify the deformation rate of the PA, the strain rate was calculated as the time derivative of the calculated PA strain. The PA tension force was calculated from the spring constants estimated based on published information and the natural lengths of the PA^42^. Details of the calculation have been previously described by Matsumoto et al.^42^. To quantify the force intensity applied to the PA, the mechanical stress, that is, the ratio of the PA force to the cross-sectional area, was calculated. For this, the PA cross-sectional area of the 19 participants was assumed to be equal to the published value^50^, 69.2 mm^2^. We then estimated the PA cross-sectional area of each participant’s foot by scaling the published value to the two-third of the body mass ratio, assuming a simple allometric relationship between body mass and PA cross-sectional area; the mechanical stress was calculated accordingly. All data were analyzed using MATLAB 2018a (The MathWorks, Natick, MA, USA).

### Statistical analysis

The kinematic and kinetic parameters at *t* = 0 (impact peak) and the four-time points were statistically compared using a *t*-test if the normality was confirmed using the Kolmogorov–Smirnov test. We used a rank-sum test for statistical comparisons if the normality was violated. All statistical tests were performed at a significance level of 5%. Regarding the PA parameters, the statistical tests were followed by Bonferroni correction for multiple testing with the adjusted p-value set at *p* < 0.01 (0.05/5). Results were presented as means and standard deviation unless otherwise stated.

## Results

### Participants

No single trial that satisfied all the above conditions was recorded for three male and four female participants. Therefore, we analyzed a total of 19 trials from ten males (27.4 ± 5.6 years, 169.3 ± 3.9 cm, and 59.6 ± 6.1 kg; mean ± standard deviation) and nine females (23.9 ± 1.1 years, 159.8 ± 6.7 cm, and 55.1 ± 8.9 kg) to identify possible sex-based differences in the kinetics and kinematics of the foot and PA during drop jump.

### Temporal parameters

The means ± standard deviations of the stance duration for females and males were 0.26 ± 0.02 s and 0.28 ± 0.01 s, respectively, which was significantly shorter in females than in males (*p* = 0.021). The time periods after the impact peak (T3 and T4) were significantly smaller in females than in males [78 ± 15 ms vs. 99 ± 19 ms for T3 (*p* = 0.006), and 142 ± 15 ms vs. 165 ± 16 ms for T4 (*p* = 0.006)], indicating that the short stance phase duration in females was due to shorter durations of the late-landing and early jumping phases.

### Ground reaction force

**Figure 3:**
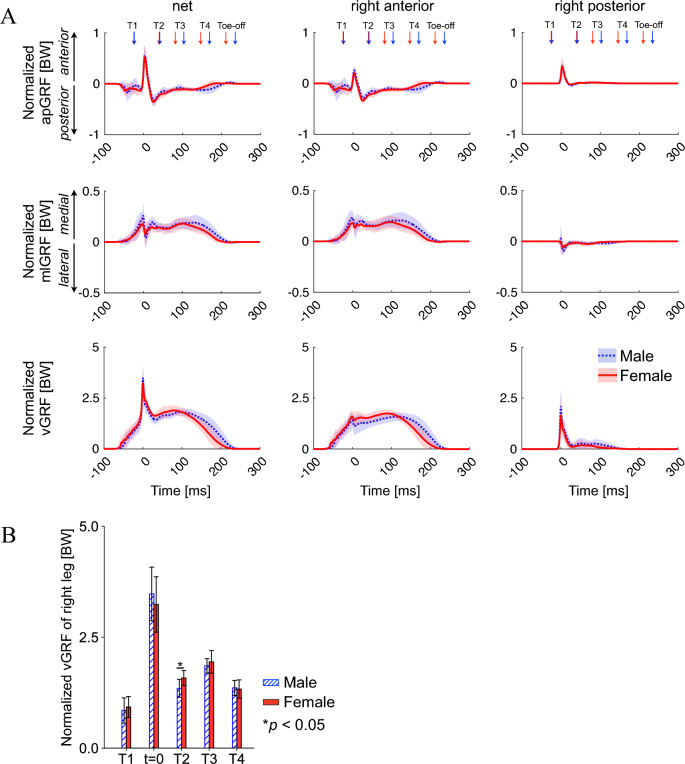
Mean ground reaction force (GRF) profiles (**A**) and comparison of the net vertical ground reaction force between sexes during drop-jumps (**B**). Mean (dotted blue line, males; solid red line, females) ± standard deviation (blue and red bands, respectively). Mean (blue box, males; red box, females) ± standard deviation (error bar). apGRF, anteroposterior GRF; mlGRF, mediolateral GRF; vGRF, vertical GRF; BW, body weight.

Vertically, the impact peak of approximately three BWs was applied at the time of heel contact (*t* = 0) in both sexes (Fig. 3A). Horizontally, the GRF was generally directed anteromedially during the heel impact but was directed posteromedially after the impact. The normalized GRF profiles were nearly identical between both sexes (Fig. 3), but vGRF was significantly larger in females than in males during the late landing phase [1.6 ± 0.2 BW vs. 1.4 ± 0.2 BW for T2 (*p* = 0.014)] (Fig. 3B).

### Joint angle

**Figure 4:**
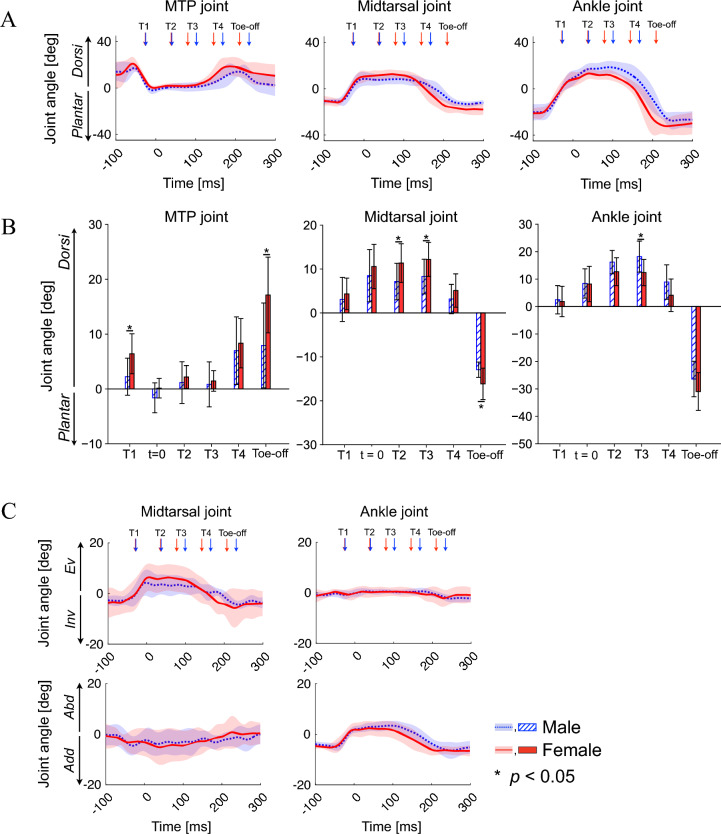
Mean foot joint angle profiles during drop-jumps. Sagittal joint angle profiles (**A**). Comparison of the sagittal joint angles between sexes (**B**). Transverse and frontal joint angle profiles (**C**). Joint angles were positive for dorsiflexion, eversion, and abduction. Dorsi, dorsiflexion; Plantar, plantarflexion; Ev, eversion; Inv, inversion; Abd, abduction; Add, adduction.

The MTP joint was plantarflexed, whereas the midtarsal and ankle joints were dorsiflexed (Fig. 4A) in the early landing phase of drop-jump (foot-contact to T2). The midtarsal joint dorsiflexion was significantly larger during the late landing phase (T2 and T3) in females than in males [11.4 ± 4.4 degrees vs. 7.1 ± 4.2 degrees for T2 (*p* = 0.047), and 12.2 ± 3.9 degrees vs. 8.3 ± 3.9 degrees for T3 (*p* = 0.044)] (Fig. 4B). The eversion of the midtarsal joint also tended to be larger in females than in males, although not to the point of statistical significance (Fig. 4C). During the jumping phase (T4 to toe-off), the MTP joint was dorsiflexed, whereas the midtarsal and ankle joints were plantarflexed (Fig. 4A). The dorsiflexion of the MTP joint at toe-off was significantly larger in females than in males [17.1 ± 6.9 degrees vs. 7.9 ± 7.7 degrees (*p* = 0.015)].

### Joint moment, angular velocity, and power

**Figure 5:**
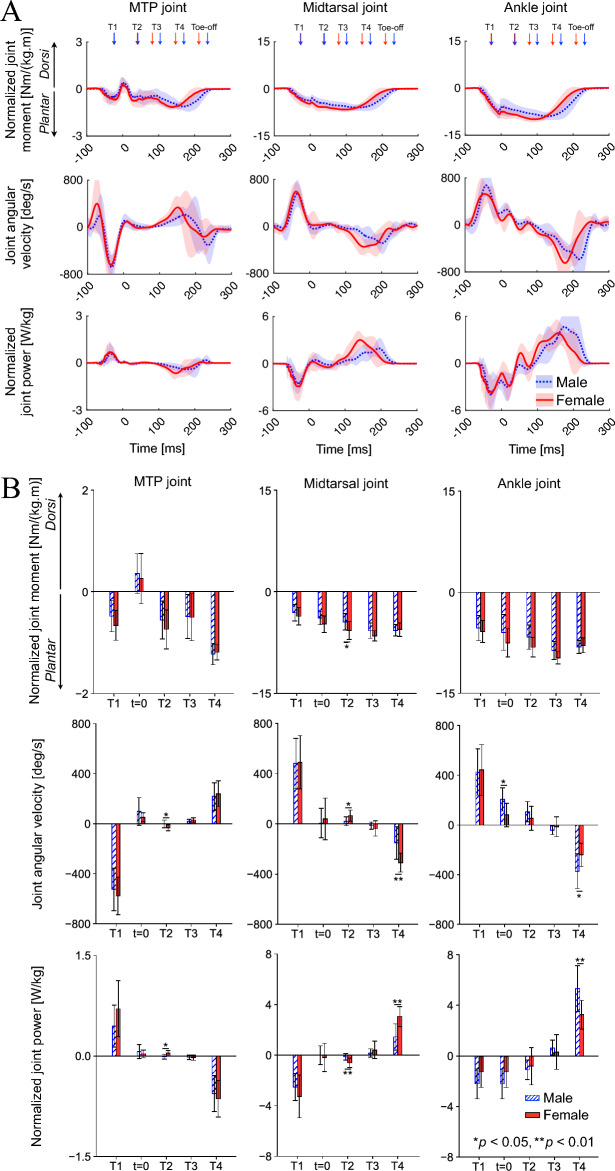
Mean foot joint moment, angular velocity, and power profiles during drop-jumps. Sagittal joint moment and power profiles (**A**). Corresponding joint angular velocity profiles are also shown. Comparisons of the moments, angular velocities, and powers between sexes (**B**). Joint moments, angular velocities, and powers were positive for dorsiflexion. The joint moments and powers in the transverse and frontal planes were not presented as they were much smaller than those in the sagittal plane.

Throughout the stance phase, the MTP, midtarsal, and ankle joints generated joint moments in the direction of plantarflexion (Fig. 5A). The normalized joint moment profiles were similar between both sexes. The MTP joint velocity was negative, whereas that of the midtarsal and ankle joints were positive in the early landing phase and vice-versa in the jumping phase. Therefore, the joint powers of the midtarsal and ankle joints were negative to absorb mechanical energy during the landing phase. However, they were positive to generate positive work to move the body upward during the jumping phase. The MTP joint power was positive and negative in the landing and jumping phases, respectively, although the magnitude was much smaller than that of the midtarsal and ankle joints.

The normalized plantarflexion moment generated around the midtarsal joint was significantly larger in females than in males during the late landing phase [5.7 ± 1.3 Nm/(kg.m) vs. 4.4 ± 1.2 Nm/(kg.m) for T2 (*p* = 0.049)] (Fig. 5B). The dorsiflexion angular velocity of the midtarsal joint was also significantly larger in females than in males [65.2 ± 45.2 degrees/s vs. 20.1 ± 35.4 degrees/s (*p* = 0.026)]. Consequently, the joint power absorbed in the midfoot during the late landing phase was significantly larger in females than in males [0.77 ± 0.5 W/kg vs. 0.17 ± 0.3 W/kg (*p* = 0.005)]. In addition, although the plantarflexion moment was not significantly different, the plantarflexion angular velocity of the midtarsal joint was significantly larger during the jumping phase [310 ± 73.2 degrees/s vs. 152 ± 129 degrees/s for T4 (*p* = 0.005)], resulting in significantly larger positive power generation by the midtarsal joint in females than in males during the jumping phase [3.6 ± 0.9 W/kg vs. 1.7 ± 1.2 W/kg (*p* = 0.002)] (Fig. 5B). However, the positive power generation by the ankle joint during the jumping phase was significantly larger in males than in females [5.3 ± 1.8 W/kg vs. 3.3 ± 1.1 W/kg (*p* = 0.010)] owing to the significantly larger magnitude of the ankle plantarflexion angular velocity [372 ± 141 degrees/s vs. 240 ± 93.3 degrees/s (*p* = 0.030)].

### Kinematics and kinetics of the plantar aponeurosis

**Figure 6:**
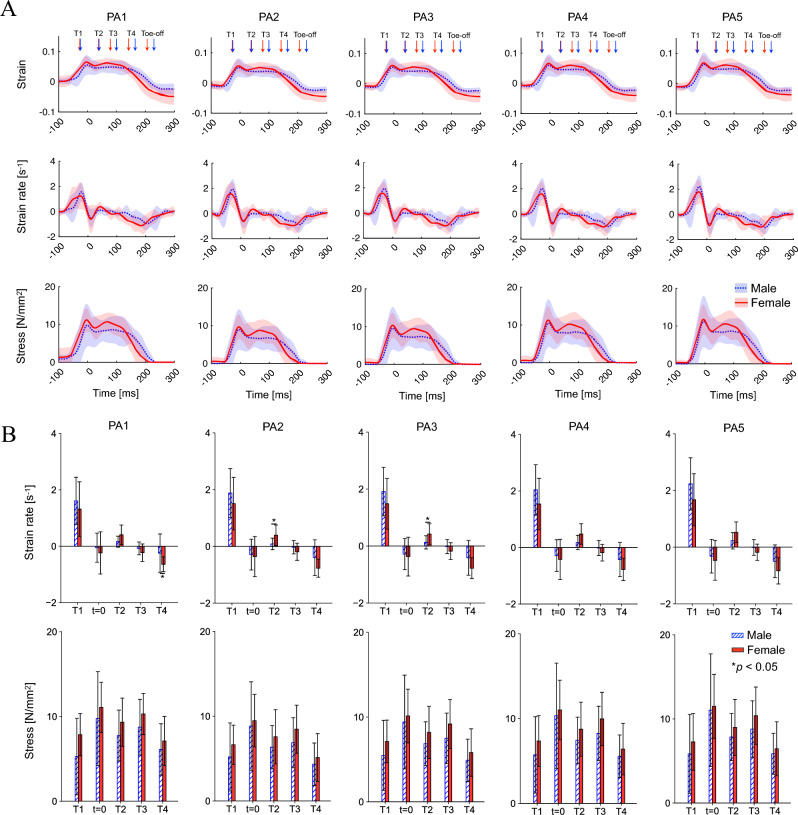
Mean PA strain, strain rate, and tensile stress profiles during drop-jumps (**A**), and comparisons of these parameters between sexes (**B**).

The PA strain profiles were generally similar between both sexes (Fig. 6A). The peak appeared just before the time of the heel-contact (*t* = 0). In females, the second peak also appeared when the whole-body COM reached the minimum vertical position (T3), but such a peak was not observed in males. The PA strain rate profiles were also generally similar between both sexes (Fig. 6A). The strain rate was the largest in the early landing phase (around T1) (Fig. 6B) in both sexes. However, the strain rate at T2 in the late landing phase tended to be larger in females than in males [0.40 ± 0.3 s^-1^ vs. 0.16 ± 0.2 s^-1^ (*p* = 0.078), 0.39 ± 0.4 s^-1^ vs. 0.09 ± 0.2 s^-1^ (*p* = 0.036), 0.42 ± 0.4 s^-1^ vs. 0.13 ± 0.2 s^-1^ (*p* = 0.049), 0.38 ± 0.4 s^-1^ vs. 0.18 ± 0.3 s^-1^ (*p* = 0.050), and 0.52 ± 0.4 s^-1^ vs. 0.22 ± 0.3 s^-1^ (*p* = 0.065) for PA1, PA2, PA3, PA4, and PA5, respectively] (Fig. 6B) due to the presence of the second peak in females. Although no statistically significant sex-based difference was found, it was close to being statistically significant. At the same time, the stress generated by the PA was also generally larger in females than in males (Fig. 6AB), although not statistically significant. Therefore, the PA strain and the stress generated by the PA tended to be larger in females than in males during the late landing phase.

#### Kinematics and kinetics of the proximal joints

**Figure 7:**
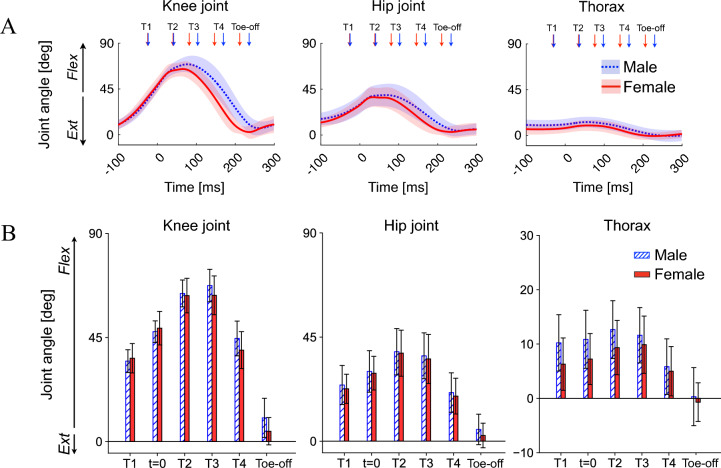
Mean joint angle profiles of the knee and hip joints and segmental angle of the trunk during drop-jumps (**A**), and comparisons of these parameters between sexes (**B**). Joint and segmental angles were positive for flexion and anterior tilt, respectively. Flex, flexion; Ext, extension.

**Figure 8:**
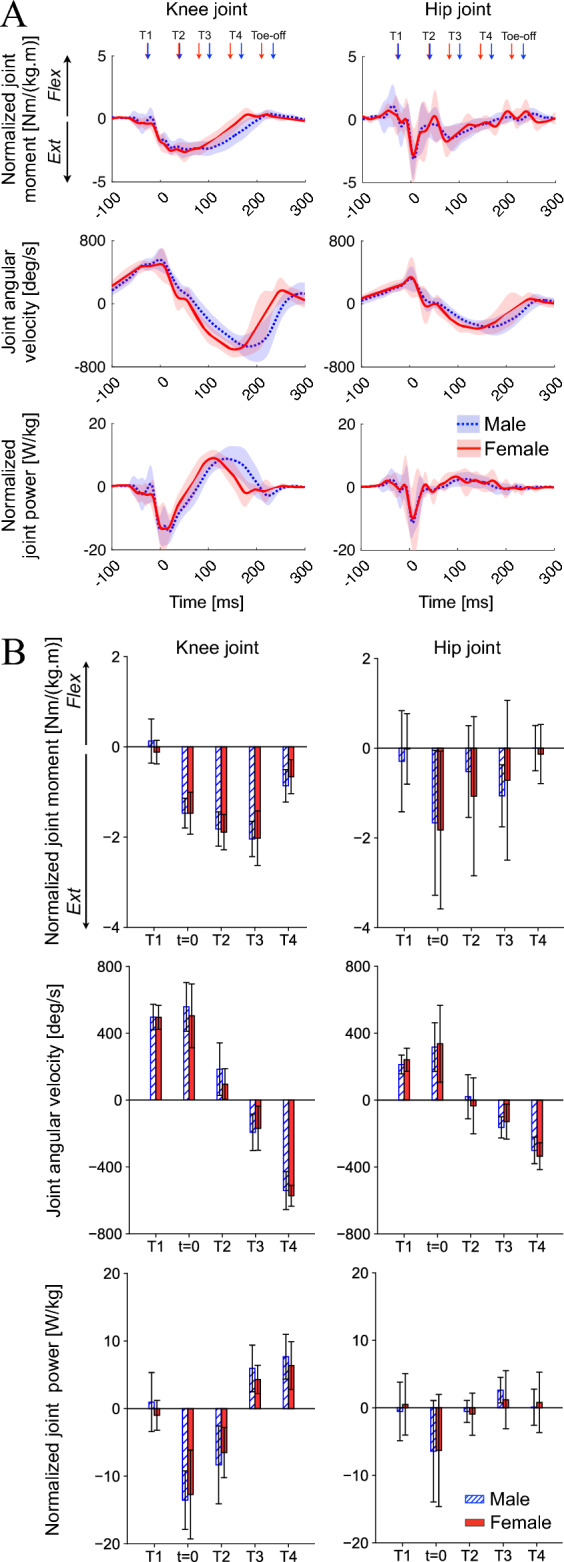
Mean joint moment, angular velocity, and power profiles of the knee and hip joints during drop-jumps (**A**), and comparison of these parameters between sexes (**B**). Joint moments, angular velocities, and powers were positive for flexion.

The knee, hip, and trunk kinematics were also generally similar between both sexes (Fig. 7AB). However, the knee was less flexed, and the trunk was anteriorly less tilted (more vertical) in females than in males during the late landing phase (T2, T3), although not statistically significant. No sex-associated differences were observed in the joint moment and power profiles of the knee and hip joints (Fig. 8A,B).

## Discussion

In this study, we investigated the differences in kinematics and kinetics of the foot and PA between sexes during drop-jump activity using a multi-segment foot model incorporating the PA. We found that some kinematic and kinetic parameters of the foot were significantly larger in females than in males, and those of the PA also tended to be larger in females than in males at the margin of statistical significance. The extracted differences in the kinematics and kinetics of the foot and PA between sexes possibly explain the higher prevalence of foot disorders in females because even small differences in the mechanical loading of the foot and PA may result in a critical difference in the possibility of developing foot disorders, including plantar fasciitis, between females and male^34^.

This study demonstrated that the midtarsal joint dorsiflexion was significantly larger in females than in males during the late landing phase. This is consistent with previous studies reporting that midfoot dorsiflexion during dynamic movements such as walking, running, and jumping was significantly larger in females than in males^30–34^. In addition, this study found that the corresponding angular velocity was significantly larger in females than in males because the stance phase duration after the impact peak was significantly shorter in females than in males. Accordingly, the strain rate of the PA tended to be larger in females than in males during the late stance phase (between the time of heel contact and the whole-body COM reached the minimum vertical position). The stress generated by the PA also tended to be larger in females than in males during the late stance phase as the elastic band connecting the heel and the toes was more extended due to greater dorsiflexion of the midtarsal joint in females than in males. Therefore, although not statistically significant, a trend was found for sex-associated differences in the PA dynamics; the PA was stretched faster and greater, and the tensile stress generated by the PA was larger in females than in males during the late landing phase. Plantar fasciitis is an overuse injury caused by excessive and repetitive mechanical stress on the PA^1,2^. The strain rate itself is the largest in the early landing phase, but the tensile stress and vertical GRF are relatively small at that time. The relatively larger stress and higher strain rate Oof the PA in females in the late landing phase after heel contact could be linked to the higher prevalence of foot injuries in females, such as plantar fasciitis.

We also found that females generated a significantly larger active plantarflexion moment and significantly larger normalized negative work around the midtarsal joint during landing. These results suggest that relatively larger forces were generated by flexor muscles of the midtarsal joint in females than in males to absorb the potential and kinetic energy of the body during landing. In this case, a larger joint contact force was possibly applied to the midtarsal joint in females than in males during landing. It has been reported that the prevalence of navicular stress fracture is higher in females than in males^51,52^. The larger plantarflexion moment and mechanical energy absorption around the midtarsal joint have been suggested to be a possible etiology or risk factor for a navicular stress fracture in females, along with their higher risk of developing osteoporosis owing to insufficient nutrition and irregular hormone levels^53^.

The midtarsal and ankle joints generated positive work to move the body upward during the jumping phase. However, we observed that females produced significantly larger positive work than males around the midtarsal joint, whereas males produced significantly larger positive work than females around the ankle joint. Therefore, there was a clear sex-associated difference in the strategy to generate work to jump upward after dropping down from a platform. The reason for this sex-associated could be attributed to the fact that the midtarsal joint mobility is significantly larger in females than in males. The differences in the foot kinematics possibly altered the kinetic strategy to generate positive power to jump upward, resulting in the difference between sexes. A larger mechanical burden was placed on the comparatively weak midtarsal joint in females and on the comparatively robust ankle joint in males. However, it must be noted that a recent study has reported that females absorbed larger mechanical energy in the ankle joint than males during landing in single-leg drop-jumps^54^, which is inconsistent with the result of this study. Further studies are necessary to clarify whether there is a clear difference in the kinematics and kinetics of the foot between sexes during dynamic movements that can be associated with the higher prevalence of foot disorders such as plantar fasciitis.

The stance duration was significantly shorter in females than in males, possibly because females tended to have more extended knees during the landing phase than males, as also noted in previous studies^55,56^. If the leg is extended more, it is structurally less compliant, and a larger vGRF is applied during landing, resulting in the shorter stance phase duration. In contrast, owing to the increased knee flexion in males, the more compliant leg possibly allows a longer time to absorb the energy and redirect the vertical movement of the COM. Because strong thigh muscles are necessary to have a more flexed knee during landing^55^, females possibly tend to extend the knee during landing, resulting in the shorter stance phase duration and larger forces and moments generated in the foot during landing. The relatively extended knee posture in females than in males were also observed in the stance phase during running^57^.

There are some methodological limitations applied to this study. First, we did not consider the possible differences in the stiffness of the PA between the sexes. The stiffness of the PA is reportedly different between sexes^58,59^. In addition, the current PA model did not consider the PA’s viscous property. Since this study found a difference in PA strain rate between sexes during the late loading phase, the PA force and stress could be much larger in females than in males if the viscous property of the PA is incorporated. For a more precise estimation of the PA forces and stresses, efforts should be made to better identify the elastic and viscous parameters necessary to quantify the stiffness and natural length of the PA. Second, the foot model used in this study did not incorporate muscles spanning the joints within the foot segment necessary for estimating the joint contact force applied to the joints. Future studies should also investigate the modeling of the paths of muscles in the foot. Third, the participants in this study were young adults who were not as physically active as regular runners. Therefore, the results of this study might not be the applicable for runners with a potential higher risk of running-related foot disorders such as plantar fasciitis. These present results should be confirmed in runners when opportunities arise in the future. Fourth, the sample size might not have effectively ensured sufficient statistical power because of the omission of the data from three male and four female participants. If the effect size was assumed to be one, the sample size of 16 for each group was necessary to achieve a power of 0.8. This could be why statistically significant differences were not detected for the PA strain rates and tensile forces between sexes. Fifth, the etiology of plantar fasciitis could be multifactorial, but the present study only focused on the biomechanical aspect of the etiology of plantar fasciitis. For example, the ability of tissue remodeling, that is, the ability of the PA to repair inflammation and micro-tears developed in the PA due to excessive and repetitive mechanical stress, could be different between sexes^60,61^ and this difference could also be another explanatory factor of the higher prevalence of plantar fasciitis in females. Establishing a more comprehensive study on the etiology of plantar fasciitis is probably necessary to provide a more complete picture of the higher prevalence of plantar fasciitis in females.

In the present study, we investigated the possible differences in kinematics and kinetics of the foot and the PA between sexes during a drop-jump activity using a multi-segment foot model incorporating the PA. Our results demonstrated that dorsiflexion and angular velocity of the midtarsal joint during the landing phase was significantly larger in females than in males. Consequently, the PA strain rate and tensile stress tended to be larger in females than in males. We suggest that differences in the kinematics and kinetics of the foot and the PA between sexes could potentially lead to a higher prevalence of foot injuries such as plantar fasciitis in females.

## Data Availability

The datasets generated and/or analyzed during the current study and the custom-made software are available from the corresponding author upon reasonable request.
